# Data on the association of the nuclear envelope protein Sun1 with nucleoli

**DOI:** 10.1016/j.dib.2017.05.028

**Published:** 2017-05-19

**Authors:** Ossama Moujaber, Nawal Omran, Mohamed Kodiha, Brigitte Pié, Ellis Cooper, John F. Presley, Ursula Stochaj

**Affiliations:** a Physiology, McGill University, Montreal, Canada; b Anatomy & Cell Biology, McGill University, Montreal, Canada

**Keywords:** Nucleus, Nucleolus, Nuclear envelope, SUN1

## Abstract

SUN proteins participate in diverse cellular activities, many of which are connected to the nuclear envelope. Recently, the family member SUN1 has been linked to novel biological activities. These include the regulation of nucleoli, intranuclear compartments that assemble ribosomal subunits. We show that SUN1 associates with nucleoli in several mammalian epithelial cell lines. This nucleolar localization is not shared by all cell types, as SUN1 concentrates at the nuclear envelope in ganglionic neurons and non-neuronal satellite cells. Database analyses and Western blotting emphasize the complexity of SUN1 protein profiles in different mammalian cells. We constructed a STRING network which identifies SUN1-related proteins as part of a larger network that includes several nucleolar proteins. Taken together, the current data highlight the diversity of SUN1 proteins and emphasize the possible links between SUN1 and nucleoli.

**Specifications Table**

**Table:** 

Subject area	*Biology*
More specific subject area	*Cell biology*
Type of data	*Fluorescence microscopy, 3D reconstruction, Western blot, sequence alignment, STRING network*
How data was acquired	*Confocal microscopy, Western blotting, database search and analysis*
Data format	*Analyzed*
Experimental factors	*Oxidative stress*
Data source location	*McGill University, Montreal, Canada and NCBI*
Data accessibility	https://www.ncbi.nlm.nih.gov/protein/

**Value of the data**

• SUN1-related proteins can localize to nucleoli.
• SUN1 nucleolar association is maintained during oxidative stress.
• SUN1 nucleolar localization is cell type specific.
• SUN1 is part of a larger network with links to the nucleolus.
• Data provide the foundation to define the mechanisms through which SUN1 controls nucleolar functions.

## 1 Data

SUN (Sad1-UNC84 homology) proteins connect the nuclear lamina to the cytoskeleton [1], [2], [3]. Most SUN proteins studied to date concentrate in the inner nuclear membrane, where they interact with other membrane components and the nuclear lamina. In the perinuclear space, SUN domains bind KASH (Klarsicht, ANC-1 and Syne homology) proteins that are embedded in the outer nuclear membrane. In this scenario, SUN proteins contain domains in the nucleoplasm, the inner nuclear membrane and the perinuclear space.

Members of the SUN protein family contribute to a wide variety of biological activities, including mechanotransduction to the nucleus [4], formation of bipolar spindles and progression through mitosis [5], DNA double strand break repair [6] and HIV replication [7]. Moreover, SUN1 and SUN2 exhibit cell-type specific functions that are critical to nucleokinesis in the developing cerebellum [8]. While there is some functional overlap between SUN1 and SUN2, both proteins make also unique contributions to cell physiology.

**Fig. 1:**
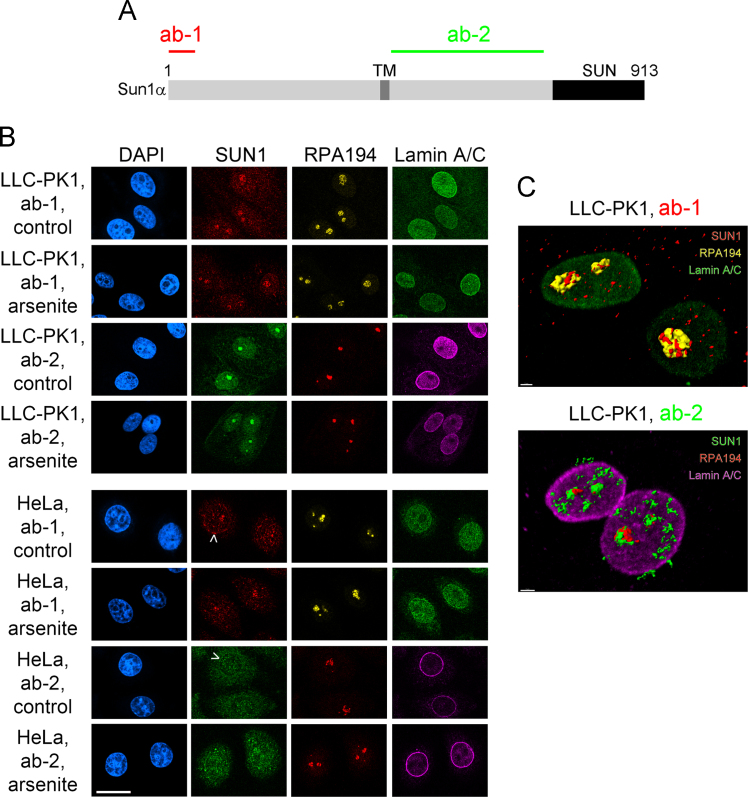
*Nucleolar association of SUN1-related proteins during control and oxidative stress conditions.* (A) A simplified model of mouse SUN1α depicts the segments recognized by antibody 1 (ab-1) and antibody 2 (ab-2, [13]). The N-terminal portion of SUN1 proteins locates in the nucleoplasm; it is followed by a transmembrane region (TM) and a segment in the perinuclear space that includes the C-terminal SUN domain. The graph was adapted and modified from [13]. (B) Indirect immunofluorescence was performed with antibodies ab-1 and ab-2 for LLC-PK1 pig kidney and HeLa cervical carcinoma cells. These antibodies were generated in different species; they recognize epitopes located in distinct segments of SUN1. Cells were grown under control conditions or treated with arsenite, fixed and stained with antibodies ab-1 or ab-2. RPA194 (RNA polymerase I, polypeptide A) provides a marker for the nucleolus; lamin A/C demarcates the nuclear lamina. Scale bar: 20 µm. Arrows point to SUN1 located at the nuclear envelope. (C) 3D reconstructions were generated with confocal stacks acquired for LLC-PK1 cells. Both ab-1 (top panel) and ab-2 (bottom) locate SUN1 proteins in nucleoli, where they are in close proximity to RPA194. HeLa cells also display weak staining of the nuclear envelope. Scale bars: 2 µm.

**Fig. 2:**
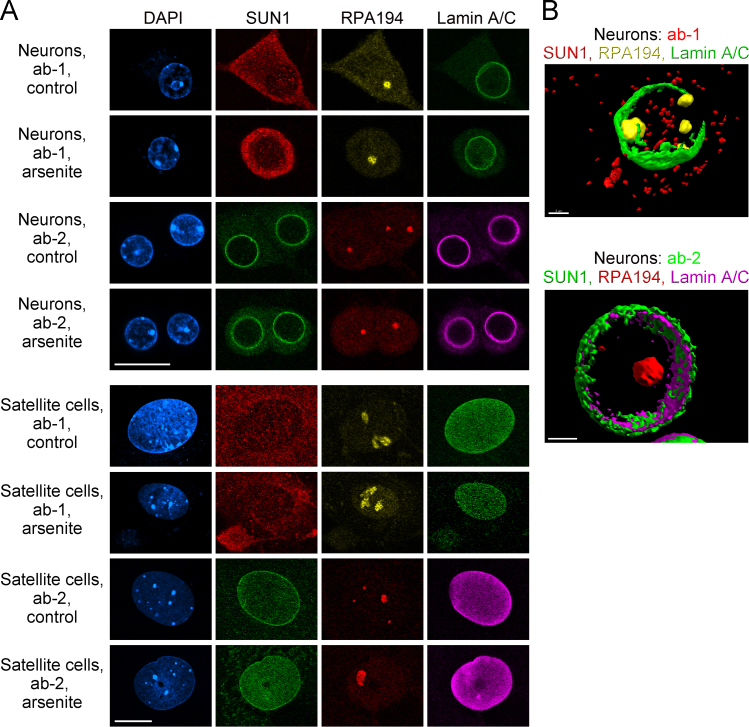
*SUN1 localization in different cell types.* SUN1-related proteins were detected in neurons and non-neuronal satellite cells of the ganglia. (A) Cells were treated and processed for immunostaining as described for Fig. 1. Scale bars: 20 µm. Note that ab-2 demarcates the nuclear envelope in neurons and ganglionic non-neuronal (satellite) cells. (B) 3D reconstructions were performed for neurons after staining with ab-1 (top) or ab-2 (bottom). Scale bars: 3 µm.

**Fig. 3:**
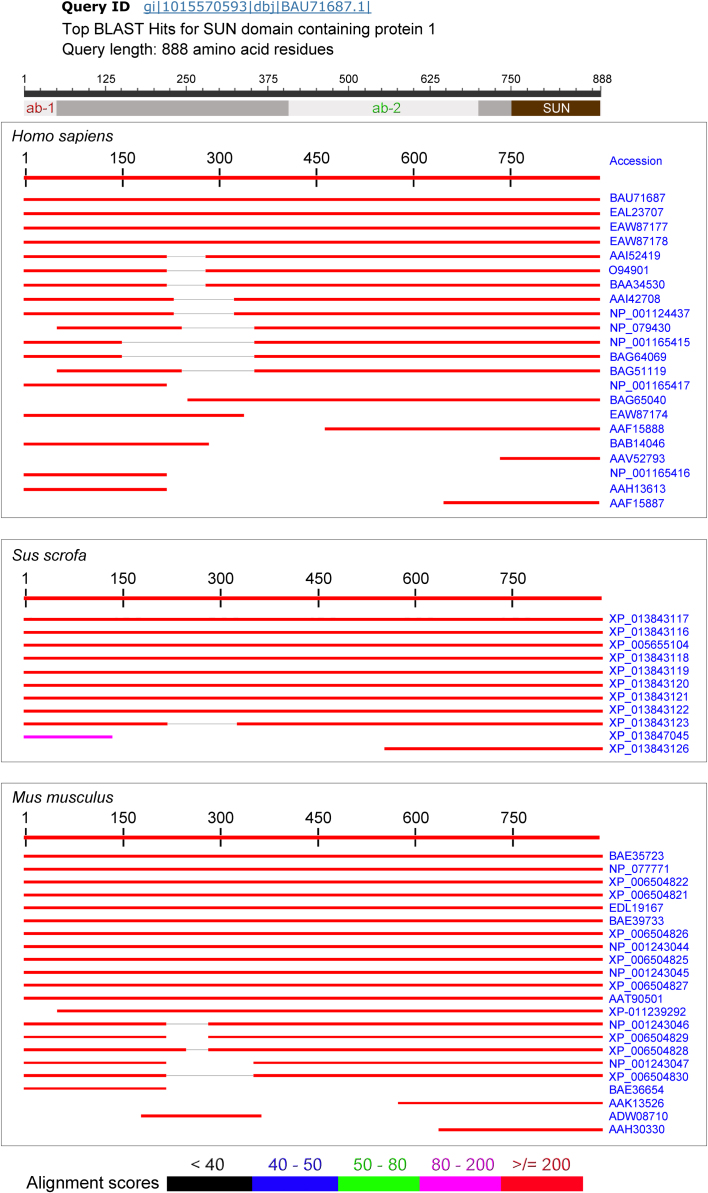
*BLAST search and alignment of SUN1 isoforms in mammalian cells*. The schematic representation on top of the figure displays the regions targeted by antibody ab-1 or ab-2 in human SUN domain-containing protein 1 (light grey) and the location of the SUN domain (brown). SUN domain-containing protein 1 was used as reference to align polypeptides with high similarity from *Homo sapiens*, *Sus scrofa* or *Mus musculus*. Only top hits are shown. They include SUN1 isoforms and UNC84-related proteins that are related to SUN1. The accession number for each hit is shown at the right margin. Note that some isoforms are missing N-terminal or internal segments, which are possibly involved in SUN1 membrane targeting.

**Fig. 4:**
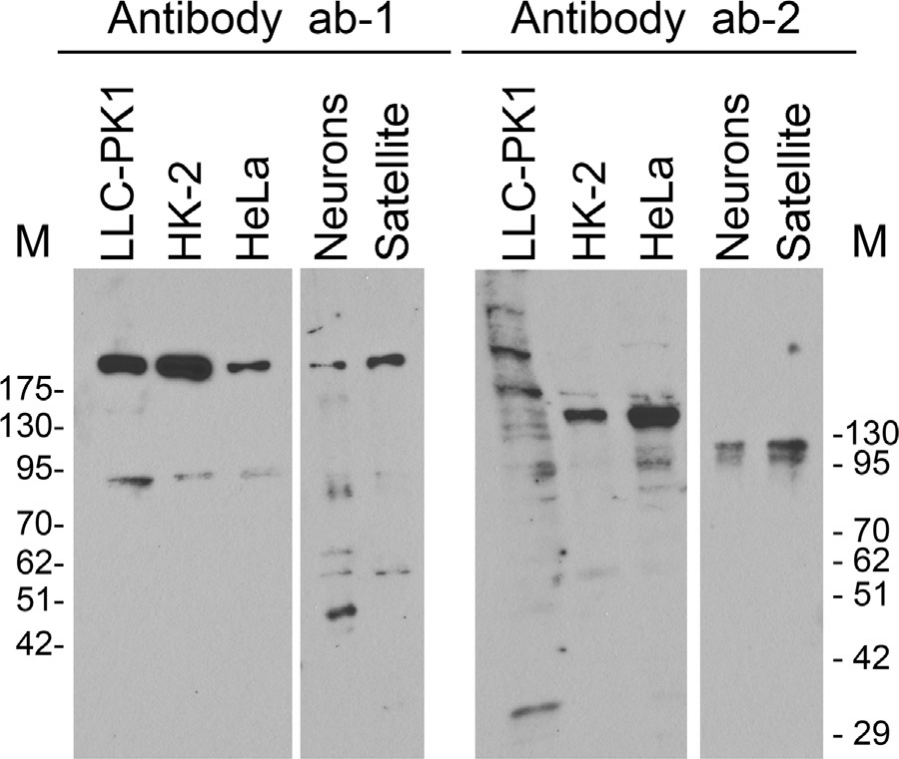
*Western blot analysis with antibodies ab-1 and ab-2.* Crude extracts prepared for LLC-PK1, HK2, HeLa, neuronal and ganglionic satellite cells were separated by SDS-PAGE and probed with ab-1 or ab-2. Molecular masses of marker proteins (kD×10^−3^) are depicted at the margins. Protein database information (Fig. 3) predicts SUN1 proteins that differ widely in their molecular mass. Indeed, Western blots in Fig. 4 show multiple bands for the cell types examined. It should be noted that numerous post-translational modifications have been identified for SUN1 [16]; this includes several ubiquitinated sites. To which extent SUN1 posttranslational modifications contribute to the complex pattern of bands is currently not known.

**Fig. 5:**
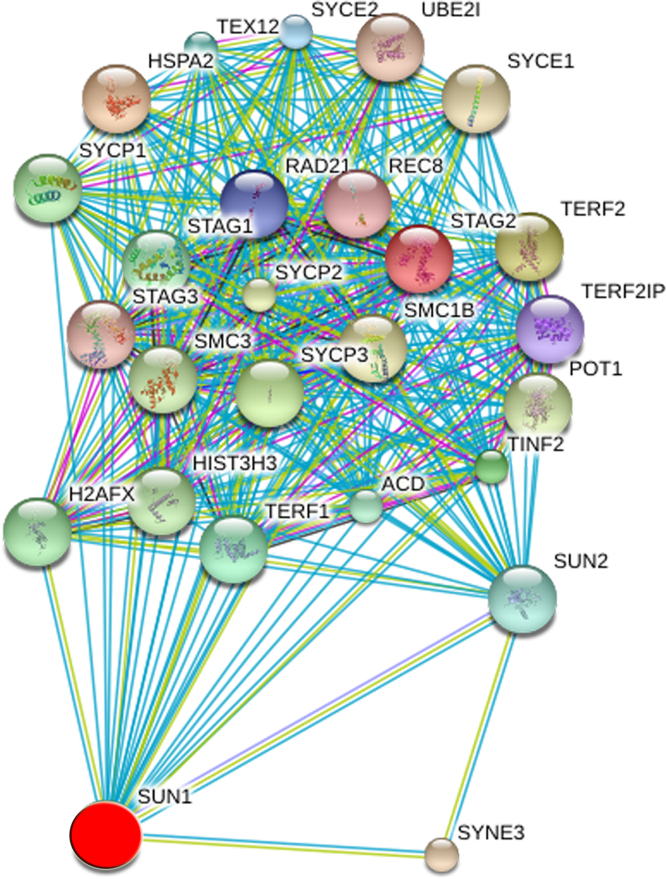
*SUN1 is part of a larger network with links to the nucleolus.* STRING network of SUN1 interacting components. The query SUN1 and first shell interactors are shown. Only components with a high confidence score ≥0.9 were included. The SUN1 network contains 26 nodes, including SUN1 and 25 different interacting components. Proteomics data for HeLa cells [17] show that SUN1 and several of its interactors have been detected in nucleoli (Supplemental File 1). For each protein, all splice isoforms are depicted as a single protein. Known and predicted interactions are included. See details in Supplemental File 1.

Our data focus on SUN1, a protein with established links to human health. For example, SUN1 promotes proper myonuclear positioning [9], and *SUN1* is a disease modifier gene for Emery–Dreyfus muscular dystrophy [9]. In addition, SUN1 can regulate adhesion to the extracellular matrix and thus affects the formation of invadopodia in cancer cells [10]. Recently, novel SUN1 activities have been described that go beyond the interaction with nuclear membranes or the lamina, suggesting that SUN1 controls nucleolar function [11], mRNA export [12] and sperm development [13]. Multiple SUN1 isoforms exist [13], [14], [15] that can differ in subcellular localization, association with binding partners and cellular function. These diverse properties of SUN1 proteins are not fully understood. Several of these properties are addressed in Fig. 1, Fig. 2, Fig. 3, Fig. 4, Fig. 5 and Table 1.

**Table 1:** Summary of data for SUN1 immunolocalization (IF) and Western blotting (WB) with ab-1 and ab-2.

**Cells**	**Ab-1**		**Ab-2**	
	**IF**	**WB**	**IF**	**WB**
LLC-PK1	**Nucleoli**	>180 kD;	**Nucleoli**	Multiple bands
		~95 kD		
HK-2	Cytoplasm, nucleus, **nucleoli**, (nuclear envelope)	>180 kD;	Cytoplasm, nucleus	~140 kD (major); additional bands of larger and smaller molecular mass
		~95 kD		
HeLa	Cytoplasm, nucleus, **nucleoli**, (NE)	>180 kD;	Nucleus, cytoplasm	~140 kD (major); additional bands of larger and smaller molecular mass
		~95 kD		
Neurons	Cytoplasm, (nucleus)	>180 kD;	NE	~100 kD (major)
		smaller bands		
Satellite cells	Cytoplasm, nucleus	>180 kD;	NE	~100 kD (major)
		smaller bands		

Supplemental File 1. SUN1 interactors identified by STRING are listed. The properties of individual nodes are described. The presence of SUN1 interactors in nucleoli is based on data published for spatial proteomics that investigated the proteome of different subcellular compartments [17].

## 2 Experimental design, materials and methods

### 2.1 Antibodies

The following antibodies were used for immunostaining at the dilutions indicated: Sun1 (BethylLab A303-438A, 1:125; ab-1) or Sun1-specific antibodies (1:50; ab-2), kindly provided by M. Alsheimer (University of Würzburg, Germany), RPA194 (Santa Cruz, sc-48385; 1:500), lamin A/C (Santa Cruz, sc-6215; 1:500). For Western blotting ab-1 and ab-2 were diluted 1:1,000. Fig. 1A shows the regions recognized by ab-1 and ab-2.

### 2.2 Cell culture

Conditions for growth of LLC-PK1 cells (renal proximal tubule, porcine) and superior cervical ganglion (SCG, mouse) neurons have been published [[18] and references therein]. HeLa (cervix adenocarcinoma, human) and HK2 (renal proximal tubule, human) cells were grown according to standard protocols. For immunostaining, mouse SCG neurons and mouse ganglionic non-neuronal cells were co-cultured and analyzed with the same methods.

### 2.3 Stress exposure

Oxidative stress was induced with 0.5 mM arsenite added in growth medium for 30 min; controls were treated with the vehicle water in growth medium.

### 2.4 Immunofluorescence

Two different methods were employed to detect Sun1 by immunostaining. Cells were either fixed with formaldehyde (ab-1) or incubated with cold methanol (ab-2), essentially as described [18]. All secondary antibodies (Jackson ImmunoResearch) were affinity-purified and pre-adsorbed to mammalian proteins to minimize non-specific binding.

In brief, for ab-1, cells were washed once in PBS, fixed in 3.7% formaldehyde/PBS for 20 min at room temperature and rinsed with PBS. Membranes were permeabilized with 0.1% Triton X-100/2 mg/ml BSA/0.1% NaN_3_ for 5 min at room temperature. Non-specific binding sites were blocked with 0.05% Tween 20, 5% FBS, 1 mM NaN_3_ in PBS for 1 hour at room temperature.

For staining with ab-2, cultured cells were washed twice with PBS. Cells were fixed and permeabilized with 100% cold methanol for 10 min at −20 °C. After rinsing with PBS, non-specific binding sites were blocked with 5% serum in PBS for 1 h at room temperature. Primary antibodies were diluted in 5% serum/PBS and cleared by 5 min centrifugation at 13,000 rpm (microcentrifuge). Samples were incubated with the supernatant overnight at 4 °C. Following two washes in PBS (15 min/wash step), affinity-purified secondary antibodies were added for 1 h at room temperature. Samples were washed three times with PBS (5 min/wash step), and nuclei were stained with DAPI.

### 2.5 Microscopy and 3D reconstruction

Image acquisition and protocols for 3D reconstruction followed standard protocols [18], [19], [20]. In brief, confocal images were acquired with a Zeiss LSM510 microscope in the multi-track mode. Filter settings were chosen to minimize cross-talk between the channels. Images were processed in Photoshop. 3D reconstructions were carried out with Imaris software.

### 2.6 Western blotting

Crude extracts were prepared, separated by SDS-PAGE and analyzed by Western blotting essentially as described [19].

### 2.7 SUN1 interaction network

SUN1 interactions were analyzed with STRING [21]. The analyses were performed at the highest confidence setting of 0.9. Proteomics data for HeLa cells have been published [17].
